# A comprehensive review on phytochemicals in the treatment and prevention of pancreatic cancer: Focusing on their mechanism of action

**DOI:** 10.1002/hsr2.2085

**Published:** 2024-04-29

**Authors:** Md. Kamrul Hasan Arnab, Md. Rabiul Islam, Mohammad Saydur Rahman

**Affiliations:** ^1^ Department of Pharmacy Jagannath University Dhaka Bangladesh; ^2^ School of Pharmacy BRAC University Dhaka Bangladesh

**Keywords:** apoptosis, autophagy, curcumin, natural products, pancreatic cancer, phytochemicals

## Abstract

**Background and Aims:**

Pancreatic cancer develops in the normal tissues of the pancreas from malignant cells. The chance of recovery is not good, and the chance of survival 5 years following diagnosis is quite low. Pancreatic cancer treatment strategies such as radiotherapy and chemotherapy had relatively low success rates. Therefore, the present study aims to explore new therapies for treating pancreatic cancer.

**Methods:**

The present study searched for information about pancreatic cancer pathophysiology, available treatment options; and their comparative benefits and challenges. Aiming to identify potential alternative therapeutics, this comprehensive review analyzed information from renowned databases such as Scopus, PubMed, and Google Scholar.

**Results:**

In recent years, there has been a rise in interest in the possibility that natural compounds could be used as treatments for cancer. Cannabinoids, curcumin, quercetin, resveratrol, and triptolide are some of the anticancer phytochemicals now used to manage pancreatic cancer. The above compounds are utilized by inhibiting or stimulating biological pathways such as apoptosis, autophagy, cell growth inhibition or reduction, oxidative stress, epithelial–mesenchymal transformation, and increased resistance to chemotherapeutic drugs in the management of pancreatic cancer.

**Conclusion:**

Right now, surgery is the only therapeutic option for patients with pancreatic cancer. However, most people who get sick have been diagnosed too late to benefit from potentially effective surgery. Alternative medications, like natural compounds and herbal medicines, are promising complementary therapies for pancreatic cancer. Therefore, we recommend large‐scale standardized clinical research for the investigation of natural compounds to ensure their consistency and comparability in pancreatic cancer treatment.

## 1 INTRODUCTION

Pancreatic cancer is a form of cancer that is characterized by the development of cancer cells that are carcinogenic within the normal tissues of the pancreas. It has become one of the leading contributors to cancer‐related deaths in almost every country in the world. According to information obtained from the Worldwide Cancer Statistics in 2012, it was listed as the seventh highest cause of death from cancer in both men and women worldwide.^1^ It was found that developed nations, such as those in Australia, East Asia, North America, and Europe, have a greater risk of pancreatic cancer.^2^ If people do not get treatment, they will have advanced cancer with a global survival time of approximately 3 months. There is just a 9% chance of recovery from this disease.^3^

At this time, adjuvant chemotherapy, immunotherapy, and surgery are thought to be the treatments for pancreatic cancer that are currently regarded as effective. The most popular way of treating pancreatic cancer is surgical resection.^4^ After the cancer has been initially identified, approximately 15%–20% of patients have been identified to be potential who could benefit from surgery. However, because of the intrusive nature of the tumor cells, approximately 80% of patients are not suitable for surgery.^5^ There is an immediate need to discover new useful medicinal medications. Since ancient times, people in China have taken up traditional Chinese medicine. Camptothecin (CPT), Podophyllotoxin (PPT), and Paclitaxel (PTX) are only a few of the natural plant medications that have been developed.^6^, ^7^, ^8^

Cannabinoids, curcumin, quercetin, resveratrol, and triptolide are some of the anticancer phytochemicals. These naturally occurring compounds are responsible for these therapeutic properties via a wide array of novel or distinctive mechanisms. Apoptosis, inhibition of metastasis, suppression of angiogenesis, and resistance are among the most recognized anticancer mechanisms. Apoptosis is a process that can also be referred to as programmed cell death (PCD), and is essential in controlling cell growth.^9^ Antiangiogenesis is the procedure of suppressing the development of additional blood vessels in already‐present blood vessels. This process can be thought of as the opposite of angiogenesis. Resistance is a process that reduces the effectiveness of anticancer medications, and permanent genetic alterations cause it frequently in cancer cells.^10^ The mechanism of action of the various naturally occurring compounds that are effective in preventing pancreatic cancer is the main aim of this review. This paper will be a valuable resource for those interested in using natural compounds for the treatment and prevention of pancreatic cancer.

## 2 METHODOLOGY

**Figure 1:**
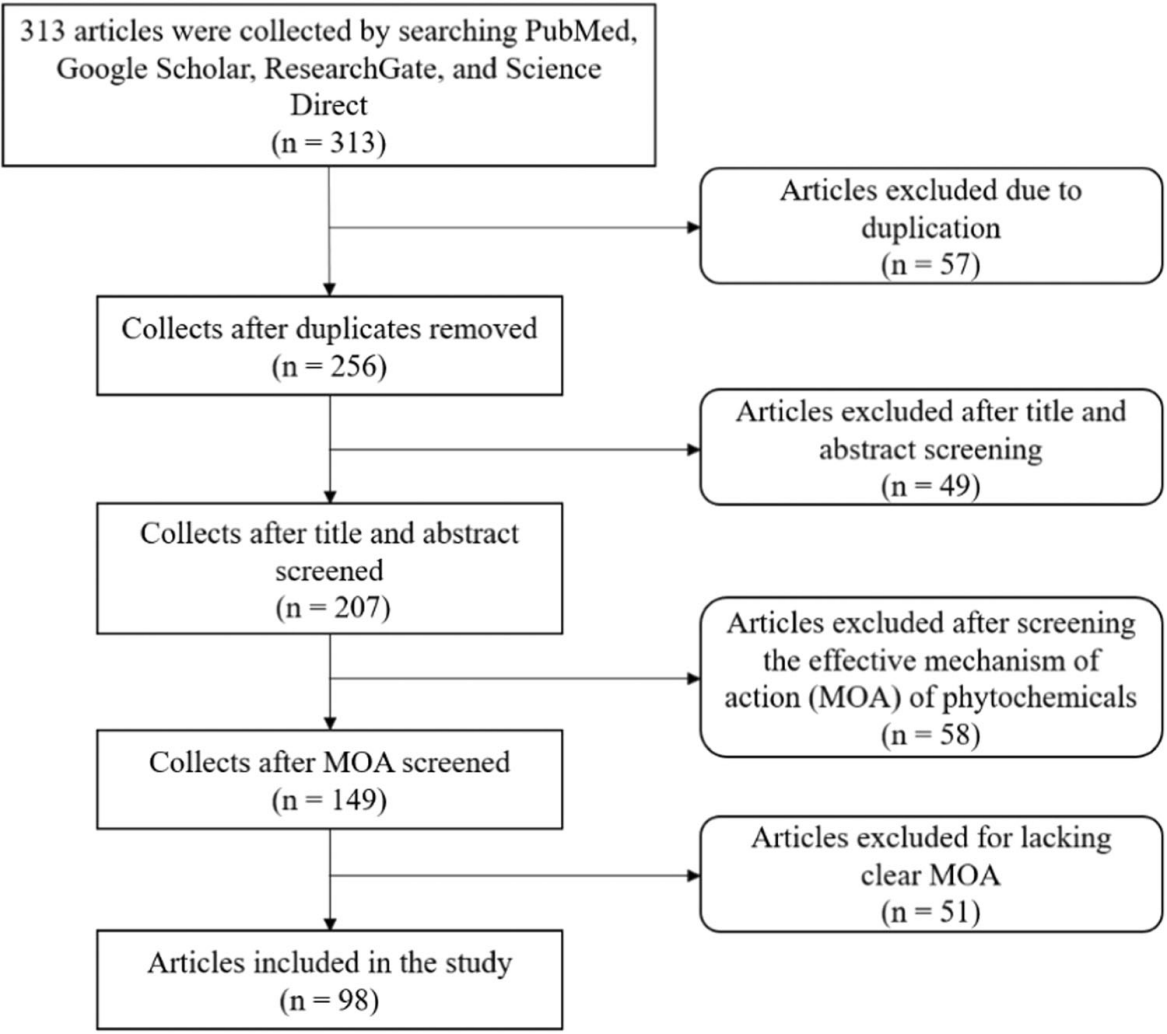
Schematic presentation of the search and selection of relevant literature.

For this review, we first enlisted some natural compounds that are used in treating pancreatic cancer. Secondly, we selected more effective phytochemicals that have the ability to prevent or suppress pancreatic cancer cell growth, focusing on their mechanism of action. The keywords were used to search for articles in various databases, including Google Scholar, PubMed, Science Direct, and ResearchGate. Articles were searched using the keywords pancreatic cancer, natural products, chemotherapeutic drugs, phytochemicals, cannabinoids, curcumin, quercetin, resveratrol, triptolide, clinical trials, apoptosis, autophagy, cell growth inhibition, cell cycle arrest, and signaling pathways, and so on. The final literature search was conducted on October 29, 2023. After reviewing everything, we decided to remove all of the duplicates. Only articles with more effective mechanisms of action and clinical trials of curcumin were considered inclusion criteria for this study (Figure 1).

## 3 PHYTOCHEMICALS FOR PANCREATIC CANCER PREVENTION

**Figure 2:**
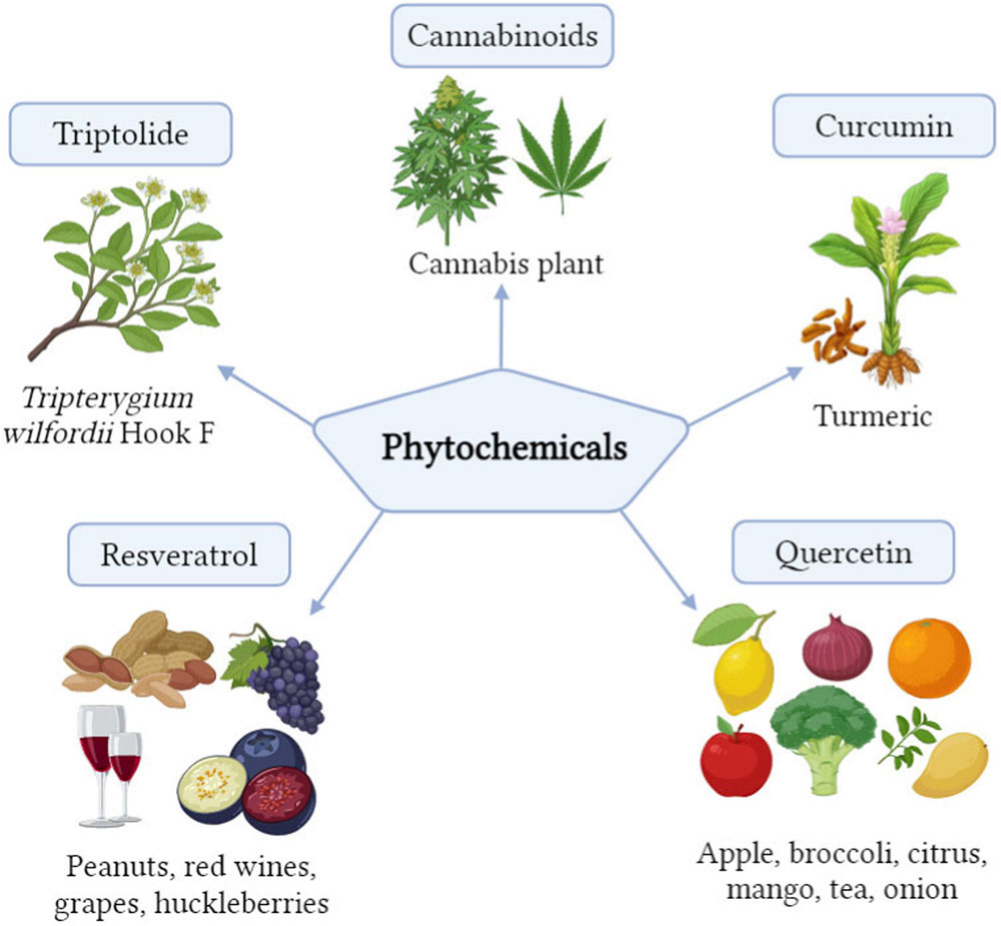
Source of phytochemicals.

Plants, animals, microorganisms, and insects all include compounds or metabolites that are known as natural products (Figure 2). Natural products and their synthetic compounds have been effective in treating cancer for a very long time.^5^ For many years, natural products played a crucial role in the research and development of anticancer medications. Several naturally occurring compounds, for example, paclitaxel and vincristine, as well as synthetic alternatives, are often utilized in clinical trials.^11^ It has been noted for being difficult to diagnose and having an adverse prognosis, despite the availability of surgical treatment and chemotherapy. Herbal medicine derived from natural products is currently being investigated for its potential as an innovative cancer treatment because of the drug's efficacy and absence of major side effects.^12^ The effectiveness of natural products and herbal medicines may be increased, as well as the adverse effects of treatment, when combined with traditional chemotherapy and radiotherapy.^13^

### 3.1 Cannabinoids

Cannabinoids are lipophilic compounds extracted from the cannabis plant that have psychoactive activity. The female flower of the cannabis plant contains the maximum number of cannabinoids. The three main categories of cannabinoids are endogenous, plant‐derived, and synthetic cannabinoids.^14^ Plant‐derived compounds are extracted from both *Cannabis sativa* and *Cannabis indica* plants.^15^ The Food and Drug Administration (FDA) has not given its approval to the therapeutic use of cannabis for the treatment of any kind of medical problem, but recent clinical studies have examined its effectiveness for side effects that are associated with cancer.^16^ Dronabinol and Nabilone are cannabinoids that are available on the market and are used for cancer‐associated side effects, including nausea, cachexia, and lethargy.^17^ Tetrahydrocannabinol (THC) and Cannabidiol (CBD) are two of the phytocannabinoids that are now getting the most clinical study from different phytocannabinoids.^18^, ^19^ They are lipophilic substances that bind directly to particular receptors for cannabinoids on the surface of cells. They stimulate various receptors like G‐protein‐coupled receptor 55 (GPR55), CB1 and CB2 for their activity.^20^ Two of the most important endogenous cannabinoids are called 2‐AG and AEA, which activate the receptors and also function on transient receptor potential ion channels such as TRPV.^20^, ^21^, ^22^

#### 3.1.1 Anticancer mechanisms of cannabinoids

Cannabinoids prohibit the growth of tumors in various ways, the most typical of which is a suppression of cell proliferation caused by apoptosis, autophagy, and cell cycle arrest.^23^, ^24^, ^25^ The messenger RNA (mRNA) contents of CB1 and CB2 receptors are either undetectable or highly reduced in healthy cells of the pancreas, whereas these receptors are present in pancreatic cancer cells.^26^ Without having any effect on healthy pancreatic cells, stimulation of the CB2 receptor may increase the ability to survive cancer cells while prohibiting apoptosis using mechanisms involving PI3K and PKB.^27^ Researchers found that ceramide has a function in the apoptosis of pancreatic cancer cells caused by THC, namely, in the increased expression of ATF‐4, p8, and TRB3 in pancreatic cancer cells.^28^ Additionally, WIN 55,212‐2 was found to trigger apoptosis in cancer cells of the pancreas by stimulating the transcription factor TRB3, which is liable for the apoptosis that is caused by endoplasmic reticulum stress.^26^ Cannabinoids can activate autophagy in pancreatic cancer cells, and it is possible that AMPK is involved in this pathway.^29^ For pancreatic cancer, the induction of reactive oxygen species (ROS)‐mediated autophagy can be accomplished by either GW405833 (GW) or arachidonoyl cyclopropamide (ACPA), which are both synthesized cannabinoid agonists appropriate for CB1 and CB2 receptors, respectively.^25^ Through the inhibition of mTORC1, the primary protein producer, and the development of cell regulators, AMPK is crucial to autophagy.^30^

**Figure 3:**
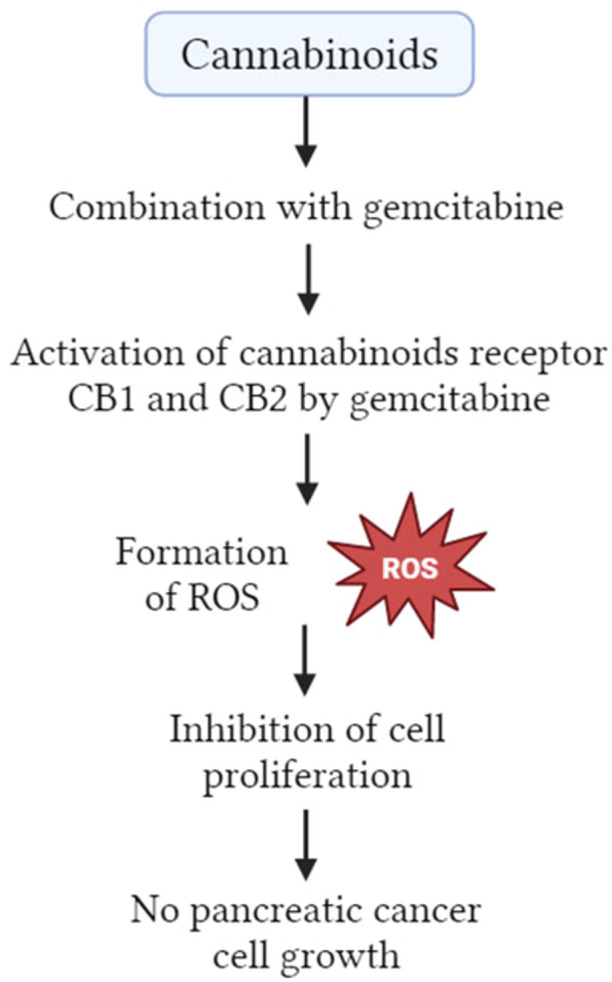
ROS‐mediated autophagy of cannabinoids and gemcitabine combination. ROS, reactive oxygen species.

In the management of pancreatic cancer, the addition of gemcitabine as well as cannabinoid receptor agonists resulted in the increased formation of ROS, which had an impact on inhibiting cell proliferation (Figure 3).^25^ Through an NF‐κB‐mediated function, gemcitabine was able to increase the expression of CB1 and CB2 mRNAs.^31^ Tumor inhibitor p53 regulates the GPR55 receptor through control of the cell cycle and the MAPK signaling systems; it may play a major function in the development of pancreatic cancer.^29^

### 3.2 Curcumin

Curcumin is a naturally occurring compound that can be derived from turmeric, which is scientifically referred to as *Curcuma longa*.^32^ Differentioloylmethane is another name for it, and an extract of it was discovered in 1815. It is indigenous not only to the Indian subcontinent but also to other tropical regions, especially Southeast Asia. It is the primary bioactive compound of turmeric and contains around 2%–5% of the total culinary herb.^33^ Herbal medicine demonstrates the efficacy of curcumin for the treatment of a variety of conditions unequivocally.^34^ Cancers including breast, stomach, lung, liver, prostate, and pancreatic have all responded effectively to its chemotherapeutic actions. Curcumin has been shown to reduce the risk of pancreatic cancer, which is one of the types of cancer that has been studied the most.^35^ The anticancer activities of curcumin have been demonstrated in quite a few preclinical investigations against cancer cells, particularly pancreatic cancer.^35^ Currently, curcumin is the only plant medication being studied for pancreatic cancer prevention in a human clinical trial. The National Cancer Institute (NCI) recognized curcumin's potential to be a third‐generation chemotherapy agent for the prevention of cancer.^36^

#### 3.2.1 Anticancer mechanisms of curcumin

**Figure 4:**
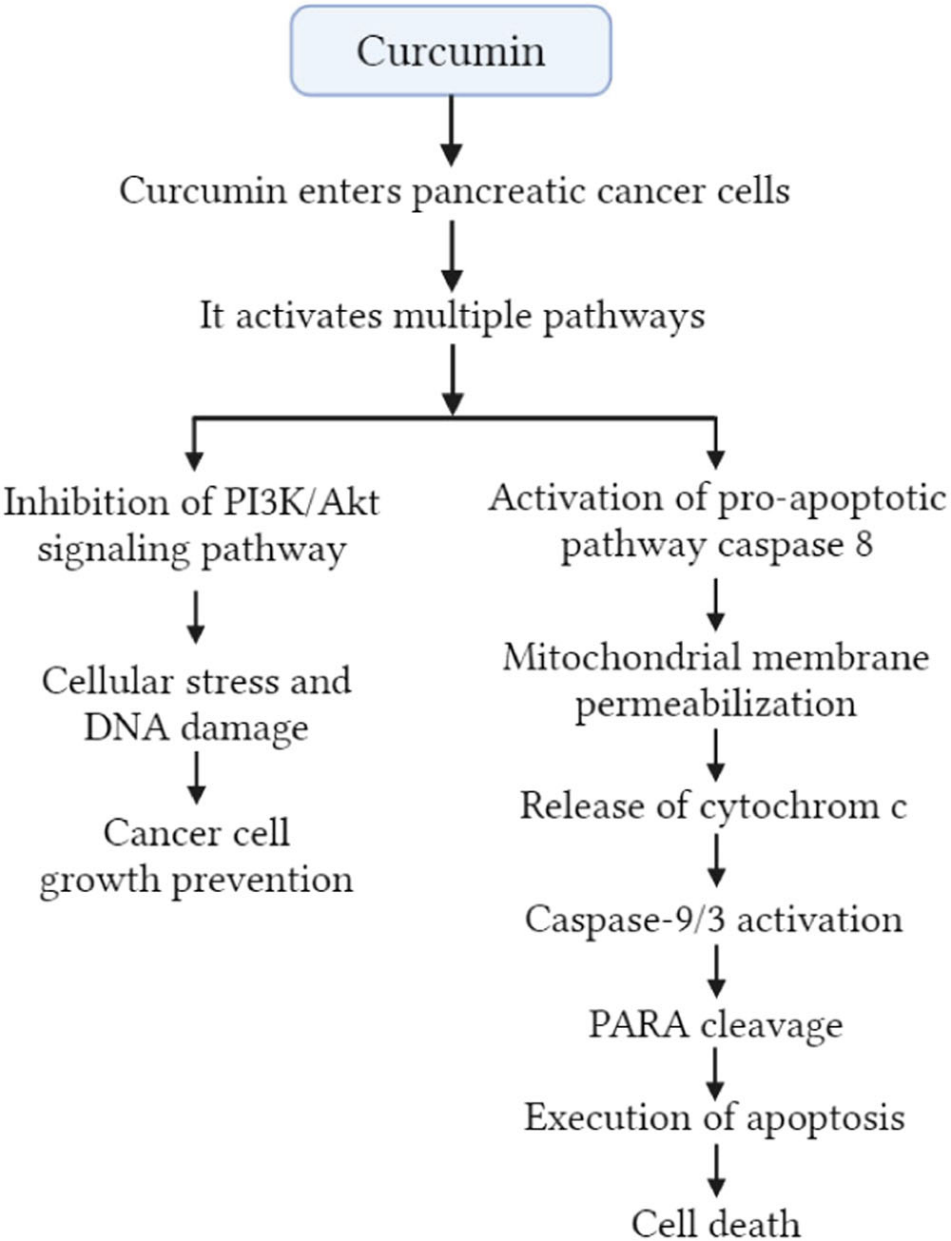
Mechanisms of apoptosis by curcumin in pancreatic cancer prevention.

Curcumin's anticancer action on pancreatic cancer is the result of various biological processes that it promotes. By suppressing NF‐κB, curcumin inhibits tumor cell development in the pancreas.^37^ By preventing the normal functioning of proteins known as inhibitors of apoptosis (IAP), curcumin was able to cause the apoptosis of pancreatic cancer cells.^38^ Through the activation of FOXO1 and the inhibitory effects in the PI3K/Akt pathway, curcumin was able to trigger programmed cell death in pancreatic cancer cells (Figure 4).^39^ The levels of expression of miR‐200, which has a vital role in controlling the epithelial–mesenchymal transformation (EMT) as well as the cancer development, can be increased by curcumin.^40^, ^41^ On the other hand, curcumin shows a tendency to reduce the function of miR‐21 that occurs in pancreatic cancer.^40^ Curcumin reduced the wound‐healing and invasion capacities of cancer of the pancreas Patu8988 and Panc‐1 cells. Curcumin caused this effect through its ability to inhibit the NEDD4/mTOR/Akt pathway.^42^ Both in vivo and in vitro, researchers found that curcumin stopped the growth of pancreatic cancer SW1990 cells and the c‐Myc antagonist 10058‐F4 in a way that works together.^43^ Through the downregulation of Sp1, curcumin was able to decrease the activity of NF‐κB as well as the proliferation of Panc‐1 and L3.6pl cancer cells.^44^ Curcumin has the ability to inhibit not only the protein phosphorylation of STAT1 and STAT3 but also the EGFR and Notch‐1 signaling processes, all of which play important roles in the progression of pancreatic cancer.^45^ Curcumin also inhibits constitutive NF‐κB via downregulating Sp1, Sp3, and Sp4 transcription factors.^39^

#### 3.2.2 Clinical trials of curcumin

The only compound or medicinal plant now being studied in clinical trials for pancreatic cancer is curcumin. A group of 25 patients who were at an advanced stage of pancreatic cancer participated in a clinical trial of phase II. In addition to other biological activities, patients were also found to have decreased levels of STAT3, NF‐κB, and COX‐2 in their peripheral mononuclear cells.^46^ Another study observed that 17 patients who were also at an advanced stage of pancreatic cancer participated in a phase II trial that studied the combined administration of curcumin and gemcitabine.^47^ A comparable curcumin plus gemcitabine‐based chemotherapy was tested in a phase I/II trial for treating gemcitabine‐resistant pancreatic cancer in 21 patients.^48^ This combination was reported to be effective. These clinical trials were performed with small participants, and researchers could not explain clear results (Table 1). Thus, extensive clinical study should be needed for understanding clear anticancer mechanisms and exact results as well.

**Table 1:** The use of curcumin in clinical studies of pancreatic cancer patients.

Therapy	Participants (*n*)	Disease condition	Dose and duration	Study	Result	References
Only curcumin	25	Advanced pancreatic cancer	8 g/day for 8 weeks	Phase II	Generally, they are well tolerated. However, absorption was poor, and it was just efficient in some of the patients. One patient had a condition that remained stable for more than 18 months, and another patient's tumor response was magnificent but only brief.	[46]
Combination of curcumin and gemcitabine	17	Advanced pancreatic cancer	8 g/day for 4 weeks	Phase II	The therapy only had a moderate effect, and it was not feasible. For gastrointestinal irritation, treatment had to be stopped for 29% of the patients.	[47]
Curcumin plus gemcitabine‐based chemotherapy	21	Gemcitabine‐resistant pancreatic cancer	8 g/day	Phase I/II	It is nontoxic and usually tolerated. Curcumin was well tolerated by all of the patients who participated in the study, and therefore none of them had to be taken out of the trial. The compliance level for oral curcumin was 100% on average. It increased median survival to 161 days, and the survival rate at 1 year was 19%.	[48]

### 3.3 Quercetin

Quercetin is a significant natural compound that is a member of the polyphenol group of flavonoids found in plants. The molecular formula of quercetin is C_15_H_10_O_7_. It is a common plant compound found in the diets of people all over the world. Vegetables, fruits, seeds, and leaves are all excellent sources of quercetin.^49^ Many types of cancer are thought to be treated or prevented with the application of flavonoids.^50^ Multiple studies, both in vitro and in vivo, have demonstrated that quercetin can prevent the formation of cancer cells. According to the findings of many studies, it is an effective treatment for several types of cancer.^51^ Quercetin, especially its synthetic compounds, has been shown to have biological functions that suppress the growth of cancer cells.

#### 3.3.1 Anticancer mechanisms of quercetin

**Figure 5:**
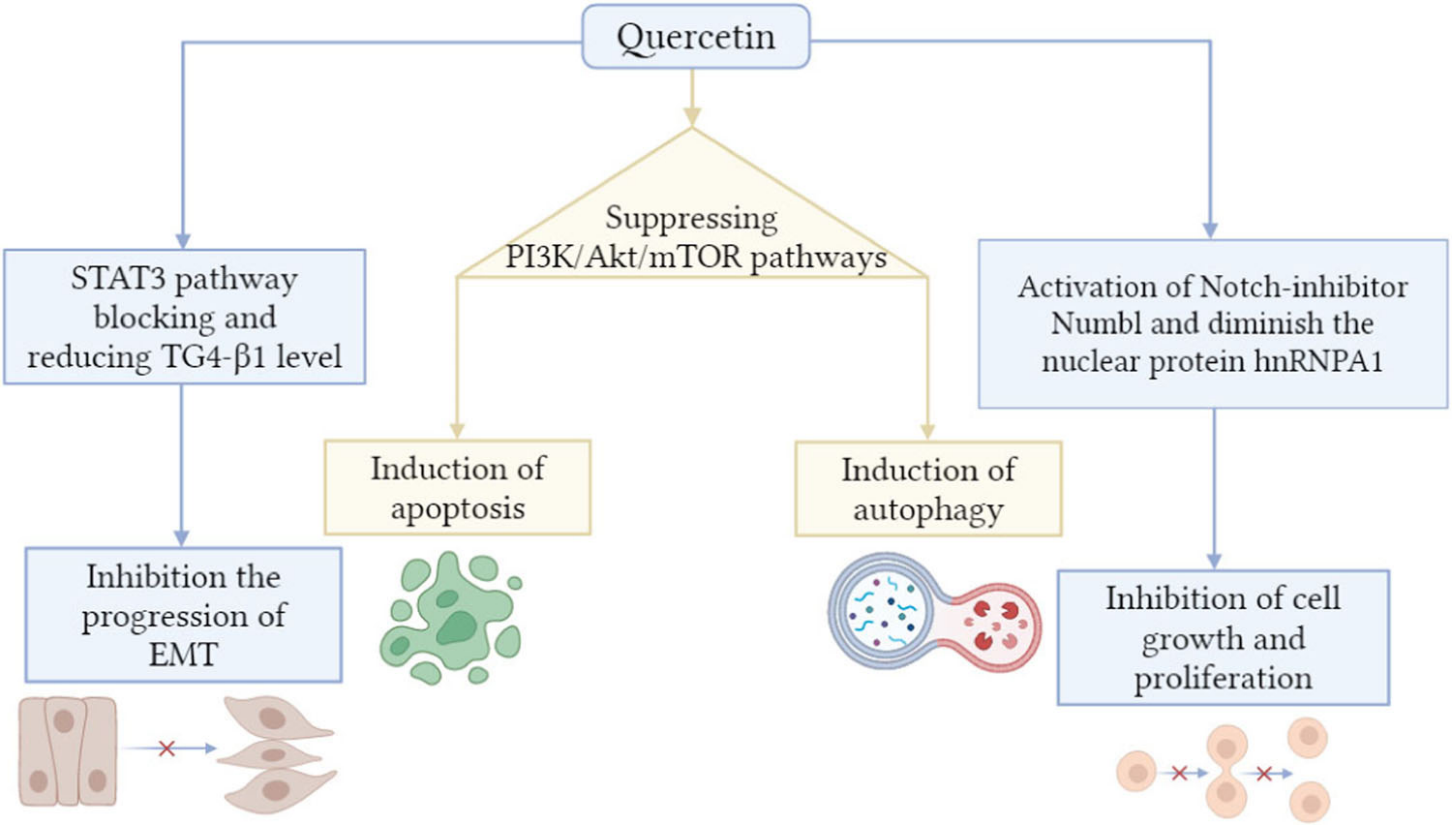
Anticancer mechanisms of quercetin. Akt, Ak strain transforming; EMT, epithelial‐mesenchymal transition; mTOR, mammalian target of rapamycin; PI3K, phosphoinositide 3‐kinase; STAT3, signal transducer and activator of transcription 3; TGF‐β1, transforming growth factor beta 1.

The anticancer properties of quercetin include the induction of autophagy and apoptosis, as well as the breakdown of the cell cycle and suppression of metastasis (Figure 5).^51^, ^52^, ^53^ It is able to have an effect on CD36 and reduce pancreatic cancer deaths in a number of different ways. Some of these ways include promoting cell adherence, improving the immune system's response, and modulating thrombospondin‐1.^54^ RAGE‐specific siRNA has caused increased autophagy and apoptosis via the suppression of the PI3K/Akt/mTOR pathways (Figure 5). To increase apoptosis and autophagy, quercetin has been demonstrated to inhibit RAGE activity.^55^ The action of quercetin on the proliferation of pancreatic cancer cells is mediated by the induction of the let‐7c target gene, which results in a reduction of cancer cell cycles and a reduction of tumor development.^56^ Quercetin also enhanced the effectiveness of BET antagonists at reducing cellular proliferation and tumor development while decreasing hnRNPA1, which regulates the translation of mRNA.^57^ It would appear that the STAT3 signaling pathway was the intended target for the anti‐EMT, anti‐invasion, and antimetastasis effects of quercetin in pancreatic cancer cells.^58^ Quercetin inhibited the activity of TGF‐ß1 as well as EMT‐TFs (snail1, zeb2) (Figure 5).^59^ It also inhibited EMT successfully, which suppressed the activity of MMP2 in addition to MMP7 and also reduced Twist2 activity.^60^ It has been shown to influence and selectively affect tumor inhibitors and carcinogenic microRNAs, along with long noncoding RNAs (lncRNAs).^61^, ^62^ The expression levels of microRNAs were reduced after being stimulated with quercetin. This was especially relevant for individuals belonging to the miR‐29, miR‐103, miR‐106, miR‐194, miR‐125, and let‐7 families. As a result, all of these microRNAs serve a significant role in inhibiting invasion, metastasis, and proliferation in addition to causing cell death, all of which are very important functions.^63^ For pancreatic ductal adenocarcinoma, quercetin inhibits the activity of miR‐200b‐3p, which results in a reduction in the activity of both Numbl and Notch.^64^

### 3.4 Resveratrol

The bioactive compound resveratrol is classified as a phyto‐polyphenol that has been isolated from many plants, including apples, black olives, capers, nuts, red rice, red grapes, and red wines.^65^ It is part of the stilbene family of chemical compounds.^66^ The roots of *Veratrum grandiflorum* were used as the initial source for its isolation in the 1940s.^67^ Resveratrol is found in high concentrations in the skin of red grapes.^68^ It comes in two different isomer types distinguished by their chemical arrangement: cis‐ and trans‐resveratrol.^69^ The accumulation of resveratrol in the cells of the plant characterizes its anti‐inflammatory and antioxidant activities.^70^ Human studies have shown that resveratrol can slow the growth of several different types of cancer cells.^71^ It has been determined that resveratrol is a crucial component in the prevention and treatment of health problems by influencing a wide range of biological pathways. It may indicate that the antioxidant capacity of resveratrol is directly related to the anticarcinogenic actions that it possesses.

#### 3.4.1 Anticancer mechanisms of resveratrol

**Figure 6:**
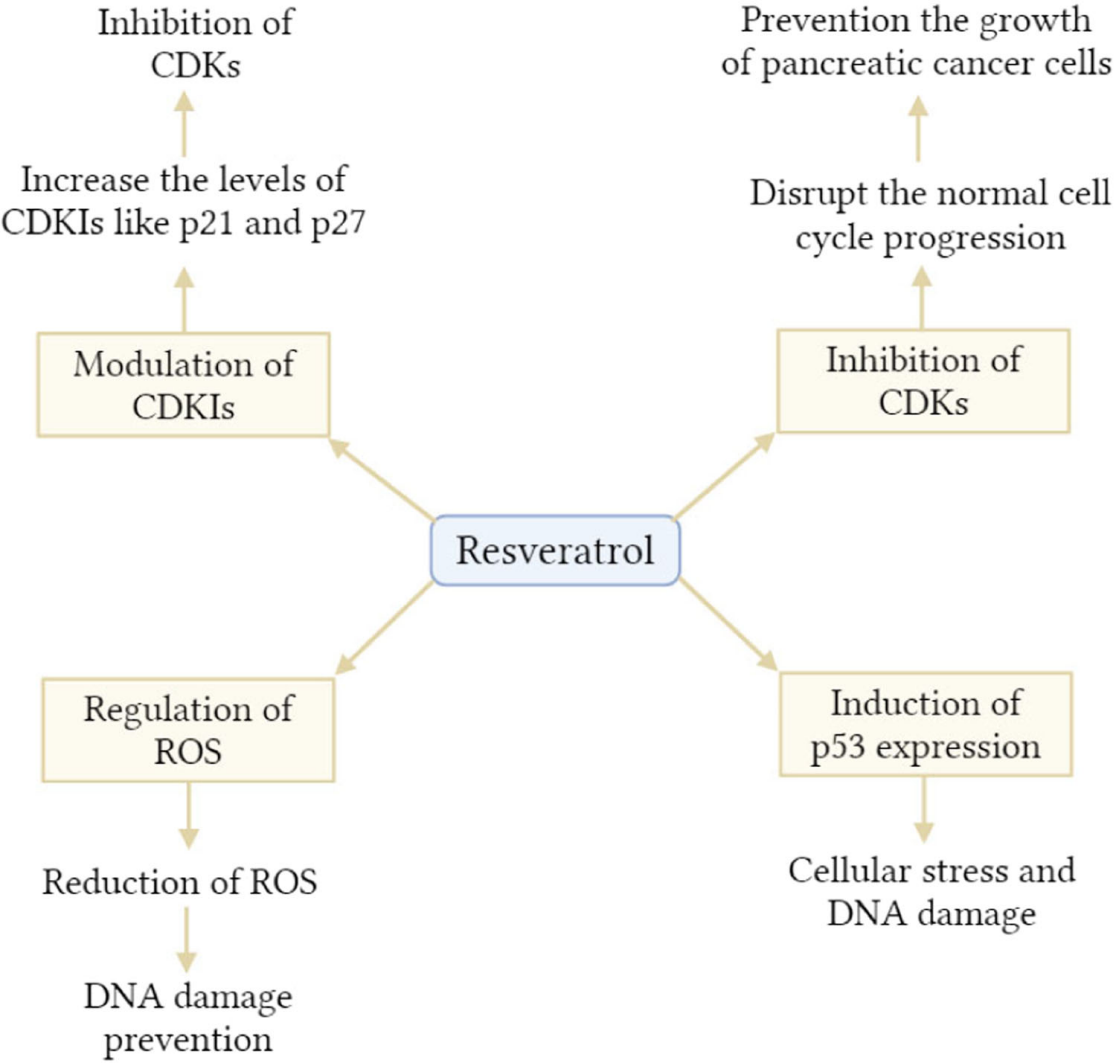
Mechanisms via which resveratrol inhibits the cell cycle in the context of pancreatic cancer prevention. CDK, cyclin‐dependent kinase; CDKIs, cyclin‐dependent kinase inhibitors; ROS, reactive oxygen species.

Because of its involvement in generating inhibition of growth, targeting a variety of signaling pathways, autophagy (Figure 6), cell cycle arrest, and apoptosis, resveratrol has been recommended as a potential treatment for preventing pancreatic cancer. Reported has been shown inhibition of AKT, NF‐κB, cyclooxygenase, cell cycle regulator, protein kinase C, matrix metalloprotease‐9, Bcl‐2 phosphorylation, focal adhesion kinase, and hydro‐peroxidase.^68^ It was discovered that resveratrol was capable of binding to LTA4H and that it could inhibit the proliferation and development of pancreatic cancer.^72^

Resveratrol can lead to apoptosis in cancer cells of the pancreas using a process that depends on mitochondria. It produces low levels of ROS, whereas these levels are considerably promoted whenever associated with ionizing radiation.^73^ For PanC‐28 and Capan‐2 cells, the INDF‐induced apoptosis and growth inhibition caused by resveratrol were considerably increased when the cells were exposed to reduced pH conditions.^74^ By blocking Akt signaling, resveratrol caused INS‐1E insulinoma cells to undergo apoptosis.^75^ Through the induction of cellular ROS formation and the activation of Nrf2 signaling, resveratrol is able to inhibit the development of NAF‐1 in pancreatic cancer cells.^76^

The impacts of resveratrol on ASPC‐1 and PanC‐1 cancer cells in the pancreas were observed to be a cell cycle arrest at the G0/G1 stage.^77^, ^78^ Reverse cell cycle arrest and a relatively low decline in viability were seen in cells treated with resveratrol without STAT3 activity but not in STAT3 activated cells.^79^ The ability of resveratrol to stop the cell cycle was found to result in greater expression of important cell cycle factors like p27/KIP1 and p21/CIP1. The same mechanism stopped the production of Cyclin D1 in pancreatic cancer cells ASPC‐1, PanC‐1, and MIA PaCa‐2.^80^ It has been demonstrated that resveratrol may suppress a number of different signaling pathways in pancreatic cancer, including FOXO, STAT3, Src, LTA4H, hedgehog, and macrophage inhibitory cytokine‐1.^81^ Resveratrol impacts the phosphorylation of FOXO in pancreatic cancer cells through the inhibition of MEK/ERK and PI3K/Akt signaling pathways.^72^

### 3.5 Triptolide

A naturally occurring compound known as triptolide has demonstrated significant potential as a potential anticancer treatment, especially for pancreatic cancer.^82^ The first known example of a diterpenoid triepoxide is called triptolide. The *Tripterygium wilfordii* Hook F (TwHF) root was used to extract triptolide in 1972.^83^ Triptolide suppresses the development of cancer cells and has demonstrated anticancer efficacy in preclinical studies of cancer of the pancreas.^84^ Triptolide is a prospective sensitizer to avoid drug resistance because its mechanism of action is distinct from conventional cancer treatments.^85^ Using low‐dose triptolide alongside other anticancer drugs is an excellent option for maximizing the efficacy against tumors and minimizing the side effects of each individual drug.^86^

#### 3.5.1 Anticancer mechanisms of triptolide

**Figure 7:**
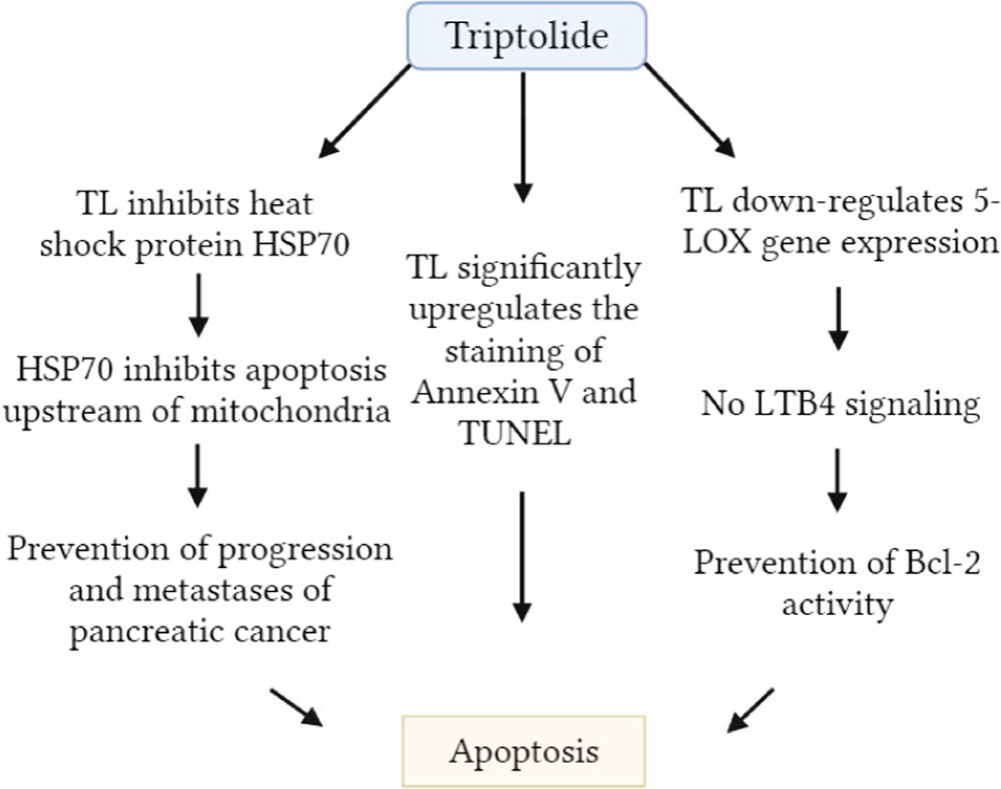
The role of apoptosis by TL in the prevention of pancreatic cancer. LTB4, leukotriene; TL, triptolide; TUNEL, terminal deoxynucleotidyl transferase.

DNA damage and apoptosis are both induced by triptolide. As a result, the expression of genes related to the repair of DNA is inhibited.^87^, ^88^ Triptolide has the ability to induce multiple distinct forms of cell death in tumor cells, including caspase‐independent cell death, apoptosis, and autophagy.^89^ However, the mechanisms of action of triptolide are not clear. The process known as apoptosis was the cause of the death of pancreatic cancer cells that were also caused by triptolide.^88^ Triptolide‐induced apoptosis occurs through a variety of pathways (Figure 7). Pancreatic cancer has a high level of expression of the heat shock protein 70 (HSP70). However, the inhibition of HSP70 by triptolide causes the apoptosis of pancreatic cancer cells.^87^ Another study reported that triptolide occurred apoptosis in the pancreatic cancer cells, which was followed by upregulation of the staining of Annexin V and TUNEL.^87^ Competitive decoy receptor 3 (DcR3) interaction is the initial step in triptolide's proapoptotic activities and is responsible for the downregulation of DcR3 in pancreatic cancer cells. Additionally, triptolide has the ability to induce upregulation of FasL and FADD, which results in the production of strong apoptotic signals.^90^ An increase in the quantity of cytochrome c and triptolide treatment both contribute to an upregulation of BAX expression in cancer cells of the pancreas, making them more susceptible to apoptosis.^90^, ^91^ The excessive expression of Bcl‐2 can be activated by an increase in LTB4, or it may be induced by 5‐LOX upregulation. Additionally, triptolide also inhibits the formation of 5‐LOX and LTB4 in pancreatic cancer cells in a manner that is dependent on both dose and duration.^92^

Triptolide promotes both apoptosis and autophagy, which leads to the death of pancreatic cancer cells. It is currently known that triptolide can enhance autophagy using a process called starvation‐induced autophagy. During the process of angiogenesis, triptolide plays an essential role in the induction of nutrient absence in pancreatic cancer cells.^93^ In pancreatic cancer cells, the protein Mcl‐1, which is a member of the Bcl‐2 family, also has a role in preventing autophagy‐induced cell death.^94^ Upregulated autophagy in pancreatic cancer cells may be caused by Ca2+, which is a proapoptotic ion.^95^ After being treated with triptolide, it was discovered that dietary starvation can induce autophagy via two different pathways: the RAF‐1/Mek‐1/ERK1/2 and the Akt/mTOR/p70s6K pathway. When triptolide was used to treat autophagy‐induction, preferring S2‐013 and S2‐VP10, phosphorylated mTOR and Akt were reduced.^95^ Compressed vasculature, impaired chemotherapy, poor prognosis, and hypoxia are the common causes of stroma in pancreatic cancer. Triptolide was studied for its potential as an antitumoral and antistromal drug in the treatment of pancreatic cancer.^96^ Reducing NF‐κB signaling is an effect of triptolide in pancreatic cancer.^97^ Triptolide is an important transcription regulator in some pathways, including ZEB1, SNAI1, and SNAI2, and related mesenchymal markers include vimentin and cadherin‐2. During this process, NF‐κB is the factor that is linked to the stimulation of hypoxia‐inducible factor 1‐alpha (HIF1‐alpha). A study reported that triptolide is able to inhibit the production of HIF1‐α as well as c‐MYC protein in pancreatic cancer cells.^98^ Molecular mechanisms of different phytochemicals to show their therapeutic effects have been presented in Table 2.

**Table 2:** Molecular mechanisms of phytochemicals.

Phytochemicals	Molecular mechanism	Mediators/pathways	Effects		References
Cannabinoids	Apoptosis	WIN 55,212‐2, ER	Endoplasmic reticulum stress stimulates the transcription factor TRB3 for apoptosis.	[26]	
		PI3K and PKB	Stimulation of the CB2 receptor may increase the ability of cancer cells to survive.	[27]	
		THC	THC activated ATF‐4, TRB3, and p8.	[28]	
	Autophagy	ACPA or GW405833	Cannabinoids also induce ROS‐mediated autophagy in pancreatic cancer.	[25]	
		AMPK	AMPK helps induce autophagy in carcinoma cells of the pancreas.	[30]	
		AMPK	Autophagy may be caused by the inhibition of mTORC1, the primary protein producer, and the development of cell regulators.	[30]	
	Cell cycle arrest	NF‐κB and Gemcitabine	Formulated ROS inhibits cell proliferation. NF‐κB‐mediated function increases the expression of CB1 and CB2 mRNAs.	[31]	
		MAPK	Tumor inhibitor p53 regulates the GPR55 receptor through control of the cell cycle and the MAPK signaling systems.	[29]	
Curcumin	Apoptosis		Inhibitor of apoptosis protein (IAP) inhibition to cause apoptosis.	[38]	
		PI3K/Akt	Curcumin was able to cause apoptosis by activating FOXO1.	[39]	
	MicroRNAs regulation		Curcumin may increase miR‐200 and decrease miR‐21 in their activity to prevent the development of pancreatic cancer.	[40, 41]	
	Invasion and migration	NEDD4/mTOR/Akt	Reduction of wound‐healing and invasion capacities of the pancreatic Patu8988 and Panc‐1 cells.	[42]	
			Curcumin inhibited the proliferation of pancreatic cancer SW1990 cells and the c‐Myc antagonist 10058‐F4.	[43]	
	Signaling pathways	STAT1, STAT3, EGFR, and Notch‐1	The STAT1, STAT3, EGFR, and Notch‐1 signaling pathways can be blocked to prevent the development of pancreatic cancer.	[45]	
Quercetin	Apoptosis and autophagy	PI3K/Akt/mTOR	RAGE‐specific siRNA inhibition	[55]	
	Cell growth and proliferation		Induction of the let‐7c target gene reduces tumor development.	[56]	
		BET	BET antagonists reduce cellular proliferation and suppress tumor development by reducing hnRNPA1.	[57]	
	Epithelial‐to‐mesenchymal transition	STAT‐3	Shows anti‐EMT, anti‐invasion, and antimetastasis effects through the signaling pathway involving STAT3.	[58]	
			TGF‐ß1, EMT‐TFs, MMP2, and MMP7 are suppressed and Twist2 activity is reduced.	[59, 60]	
	MicroRNAs regulation		Downregulates the miR‐29, miR‐103, miR‐106, miR‐194, miR‐125, and let‐7 expressions.	[63]	
Resveratrol	Apoptosis		Apoptosis is caused by high levels of ROS produced by resveratrol and ionizing radiation.	[73]	
		Akt	Apoptosis of INS‐1E insulinoma cells	[75]	
	Autophagy	ROS, Nrf2	Resveratrol inhibits the development of NAF‐1.	[76]	
	Cell cycle arrest	STAT3	The expression of key cell cycle components increased with cell cycle arrest, and Cyclin D1 was suppressed in pancreatic cancer cells.	[80]	
	Signaling pathways	MEK/ERK and PI3K/Akt	Resveratrol may prevent pancreatic cancer development by inhibiting various signaling pathways.	[72, 81]	
Triptolide	Apoptosis		Triptolide inhibits HSP70 protein to cause apoptosis in pancreatic cancerous cells.	[87]	
			Triptolide caused apoptosis in pancreatic carcinoma cells by following the staining of Annexin V and TUNEL.	[87]	
			Triptolide downregulates DcR3 and upregulates FasL and FADD for the production of strong apoptotic signals.	[90]	
			Triptolide inhibits the formation of 5‐LOX and LTB4 in pancreatic cancer cells.	[92]	
	Autophagy	Mcl‐1	Mcl‐1 protein prevents autophagy‐induced cell death in pancreatic cancer.	[94]	
		Ca2+ ion	Proapoptotic Ca2+ ion upregulates autophagy in pancreatic cancer cells.	[95]	
		RAF‐1/Mek‐1/ERK1/2 and Akt/mTOR/p70s6K	Dietary starvation can induce autophagy through these different pathways.	[95]	
	Antistromal and antitumoral activity		Triptolide shows antistromal activity by reducing the dense stroma from the cancerous cells in the pancreas.	[96]	
	Mesenchymal transition and metastasis	NF‐κB	HIF1‐α is activated during NF‐κB signaling. Triptolide can inhibit the activation of HIF1‐α and c‐MYC proteins by reducing NF‐κB signaling.	[98]	

## 4 CONCLUSION

Phytochemicals seem to have encouraging potential for preventing pancreatic cancer by inhibiting or decreasing the development of cancer cells. More comprehensive and long‐term clinical investigations are necessary to comprehend the role of these natural compounds in pancreatic cancer prevention and their chemoprevention mechanisms. Curcumin is the only compound or medicinal plant now being investigated in clinical trials for pancreatic cancer prevention. However, the appropriate dosage, clinical trial, safety profile, and effectiveness of phytochemicals in preventing pancreatic cancer all need more study. So, it is essential to conduct additional clinical trial research to investigate the function that these compounds play in the treatment and control of cancer as well as their actual mechanisms in the prevention of pancreatic cancer.

## AUTHOR CONTRIBUTIONS

**Md. Kamrul Hasan Arnab**: Data curation; investigation; methodology; software; writing—original draft. **Md. Rabiul Islam**: Conceptualization; supervision; writing—review and editing; visualization. **Mohammad Saydur Rahman**: Conceptualization; investigation; methodology; project administration; supervision; visualization; writing—review and editing.

## CONFLICT OF INTEREST STATEMENT

The authors declare no conflict of interest.

## TRANSPARENCY STATEMENT

The lead author Md. Rabiul Islam, Mohammad Saydur Rahman affirms that this manuscript is an honest, accurate, and transparent account of the study being reported; that no important aspects of the study have been omitted; and that any discrepancies from the study as planned (and, if relevant, registered) have been explained.

## Data Availability

Data sharing is not applicable to this article as no data sets were generated or analyzed during the current study.
